# Biological Tissue-Inspired Ultrasoft, Ultrathin, and Mechanically Enhanced Microfiber Composite Hydrogel for Flexible Bioelectronics

**DOI:** 10.1007/s40820-023-01096-4

**Published:** 2023-05-28

**Authors:** Qiang Gao, Fuqin Sun, Yue Li, Lianhui Li, Mengyuan Liu, Shuqi Wang, Yongfeng Wang, Tie Li, Lin Liu, Simin Feng, Xiaowei Wang, Seema Agarwal, Ting Zhang

**Affiliations:** 1 i-Lab, Nano-X Vacuum Interconnected Workstation, Key Laboratory of Multifunction Nanomaterials and Smart Systems, Suzhou Institute of Nano-Tech and Nano-Bionics (SINANO), Chinese Academy of Sciences (CAS), Suzhou, 215123 Jiangsu People’s Republic of China; 2 Department of Chemistry, Bavarian Center for Battery Technology (BayBatt), Bayreuth Center for Colloids and Interfaces, and Bavarian Polymer Institute, Macromolecular Chemistry II, University of Bayreuth, Universitätsstrasse 30, 95440 Bayreuth, Germany; 3 School of Nano-Tech and Nano-Bionics, University of Science and Technology of China, Hefei, 230026 Anhui People’s Republic of China; 4 Center for Excellence in Brain Science and Intelligence Technology, Chinese Academy of Science, Shanghai, 200031 People’s Republic of China

**Keywords:** Fiber, Hydrogel, Flexible electronics, Thin film, Electrospinning

## Abstract

Hydrogels offer tissue-like softness, stretchability, fracture toughness, ionic conductivity, and compatibility with biological tissues, which make them promising candidates for fabricating flexible bioelectronics. A soft hydrogel film offers an ideal interface to directly bridge thin-film electronics with the soft tissues. However, it remains difficult to fabricate a soft hydrogel film with an ultrathin configuration and excellent mechanical strength. Here we report a biological tissue-inspired ultrasoft microfiber composite ultrathin (< 5 μm) hydrogel film, which is currently the thinnest hydrogel film as far as we know. The embedded microfibers endow the composite hydrogel with prominent mechanical strength (tensile stress ~ 6 MPa) and anti-tearing property. Moreover, our microfiber composite hydrogel offers the capability of tunable mechanical properties in a broad range, allowing for matching the modulus of most biological tissues and organs. The incorporation of glycerol and salt ions imparts the microfiber composite hydrogel with high ionic conductivity and prominent anti-dehydration behavior. Such microfiber composite hydrogels are promising for constructing attaching-type flexible bioelectronics to monitor biosignals.

**Supplementary Information:**

The online version contains supplementary material available at 10.1007/s40820-023-01096-4.

## Highlights

- A novel strategy was developed to construct ultrathin microfiber composite hydrogel films (< 5 μm) by embedding an electrospun fiber network into a hydrogel.
- The microfiber composite hydrogel offers tunable modulus in a broad range (from ~ 5 kPa to tens of MPa), which matches the modulus of most biological tissues and organs.
- The ultrathin configuration and ultrasoft nature allow the microfiber composite hydrogel seamlessly attaching to various rough surfaces.

**Supplementary Information:**

The online version contains supplementary material available at 10.1007/s40820-023-01096-4.

## Graphical abstract

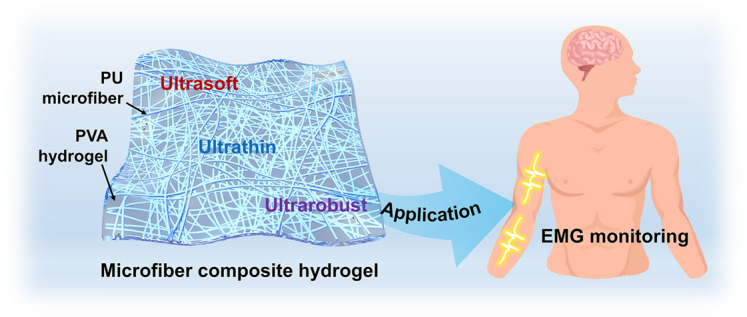

## Introduction

Wearable and implantable bioelectronics enable a variety of applications in personalized healthcare monitoring and precision medicine, offering a range of capabilities including biosignal detection, health monitoring, nerve stimulation, brain-computer interfaces, neuromuscular junctions, and cyborg tissues [1–8]. Since most of the human tissues are inherently soft (modulus in a range of 1 kPa to 1 MPa), electronic devices based on metals, ceramics, and plastics present dry, nonliving, rigid, and fragile features, resulting in striking mismatches in tissue–electrode interfaces regarding mechanics, biosignal transmission, and biological natures [9–11]. In particular, the mechanical mismatch between electronics and tissues not only affects the device's performance but also causes severe damage to the local tissues [12]. Narrowing the mechanical mismatch between tissue and bioelectronics is essential for these issues [13]. Additionally, biological tissue conducts electricity generally using ions, which differs from electron transmission mostly used in electronics. However, ionic and electronic circuits are coupled at tissue–electrode interfaces in the electrophysiological study of living human tissues [10, 14]. Therefore, bioelectronics is supposed to function through distinct ionic and electronic circuits.

The hydrogel is a network composed of hydrophilic polymers that contain a high amount of water and thus present inherent physicochemical similarities with natural biological tissue, offering a great potential candidate for engineering tissue scaffolds with high bioaffinity [15–17]. The polymer network endows the hydrogel with mechanical elasticity, and the water molecule and soluble salt ions endow the hydrogel with ionic conductivity [18]. Generally, the hydrogel possesses a high water content of over 90 %, which endows them with excellent softness, however, weakens its mechanical strength (a few kilo Pascals or even lower) [19]. Hydrogels with low mechanical strength are not easy to use as a self-standing thin film (< 10 μm) [20]. Current bioelectronic-utilized hydrogels often have a large thickness of a millimeter-scale or even larger, which leads to a broad gap between the electrode and biological tissue [21, 22]. Particularly for healthcare monitoring applications, intimate contact between sensing components and the biological tissues would effectively reduce contact impedance, minimize motion artifact, enhance measurement accuracy, and simplify subsequent data-possessing algorithm [23–27]. Recently, efforts for thin hydrogel films are widely explored by using cast-molding [28–30], blade-coating, or spinning coating [31] methods, but the thickness yet remains several micrometers and even larger. Still, it is yet very challenging to fabricate ultrathin hydrogel films with controlled micro-scale thickness and excellent mechanical properties.

**Fig. 1:**
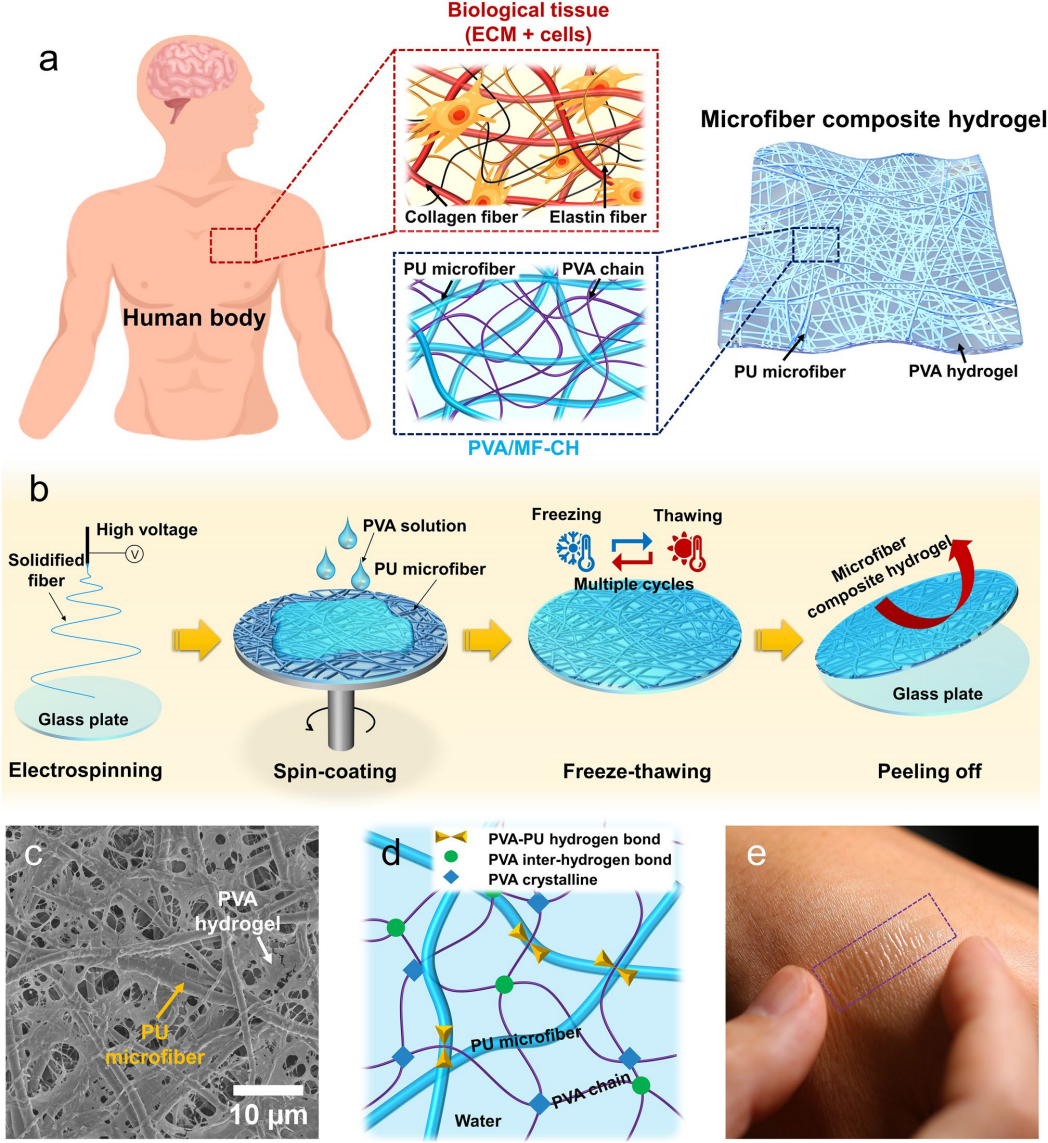
Design of PVA/MF-CH-based bioelectronics. **a** Schematic structure of biological tissue and PVA/MF-CH. In the human body, the ECM of living tissues mainly comprises of interpenetrating collagen fiber and elastin fiber networks. The PVA/MF-CH presents an ECM-mimicked structure of interpenetrating networks, which consists of PU microfibers (blue) and PVA molecular chains (purple). The PVA hydrogel comprising PVA chains and water are presented in semi-transparent blue. **b** Schematic preparation procedure of PVA/MF-CH. **c** SEM image displaying the surface of a freeze-dried PVA/MF-CH. **d** Schematic illustration of the microfiber embedded structure and bonding mechanisms of PVA molecular chains and PU microfiber matrix. **e** Digital picture of PVA/MF-CH attached to the skin

Mechanical properties of hydrogels can be improved by making designed architecture inspired by the extracellular matrix (ECM) which serves as the scaffold for tissues and organs throughout the body, playing an essential role in their structural and functional integrity [32, 33]. The natural ECM (Fig. 1a) is comprised of a stiff collagen fibril scaffold embedded in the elastic interwoven elastin matrix, which imparts biological tissues with very high fracture toughness and excellent softness [34–36]. Inspired by the fibril network structure of the natural ECM, a variety of fibers, including ceramic, natural cellulose, and various polymeric fibers are extensively explored to fabricate composite hydrogels with enhanced mechanical properties [37–41]. For example, a polyurethane (PU) nanofiber-embedded hybrid hydrogel [42] achieved enhanced softness (modulus ∼1.8 MPa) and stiffness (37 times enhancement), excellent stretchability (680 %), and fatigue resistance (∼2950 J m^−2^). However, such hydrogels generally are yet too thick to realize the intimate contact when used as attachable electronics, resulting in a decrease in the device’s performance.

In this work, we mimic the fibril structure of the natural ECM to develop an ultra-flexible, ultrathin, and mechanically enhanced microfiber composite hydrogel by embedding an electrospun polyurethane microfiber network into the poly(vinyl alcohol) (PVA) hydrogel. Such a hybrid system leads to tunable thickness, Young’s Modulus, and mechanical strength in a wide range by precisely tailoring the physiochemical properties of the fiber skeleton and the hydrogel. A composite ultrathin hydrogel film (thickness < 5 μm) was successfully prepared by combining two different processing methods: electrospinning and spin-coating. The broadly tunable Young’s Modulus of our microfiber composite hydrogels imparts a prominent mechanical match with most human living organs and tissues, endowing them with a high promise for wearable and implantable bioelectronic devices. Additionally, the incorporation of specific substances (such as sodium chloride (NaCl) and glycerol (Gly)) imparts the composite hydrogel enhanced ionic conductivity, anti-freeze, and anti-dehydration properties, which are significant for epidermal electronics to adapt to complex environments. At last, we demonstrate a high-performance flexible electrode to monitor electromyography (EMG) biosignals for a long time. Our work provides a facile solution to design and fabricate ultrathin bioelectronics for seamless attachment to soft biological tissues for healthcare monitoring applications, which will promote the progress of thin film-based bioelectronics for wearable and implantable purposes.

## Experimental and Calculation

### Materials

PU (product no. A85P4394, Mw = 70,000, density = 1.12 g cm^−3^) was purchased from Shanghai Huntsman Polyurethanes Specialties Co., Ltd., China; NaCl (Analytical purity) and N, N—Dimethylformamide (DMF, 99.5 %) were purchased from Sinopharm Chemical Reagent Co., Ltd., China; glycerol (MW 92.09, 99.0 %) and PVA (1799, alcoholysis degree 98 %–99 %) were purchased from Shanghai Titan Scientific Co., Ltd., China. Deionized water was prepared with Milli-Q equipment (Merck, Germany).

### Preparation of PVA/MF-CH and PVA/MF/Gly-CH

PU microfiber networks were prepared by electrospinning 22 wt% PU solution (in DMF) and collected with a glass plate. The electrospinning parameters are as follows: voltage 12 kV, feeding rate of PU solution 0.1 mm min^−1^ (within a 5 mL syringe), and collecting distance between the needle and the grounded collector 15 cm. The electrospinning times of 40 s, 2 min, 5 min, and 8 min were used to prepare the microfiber networks in different fiber densities. Next, the glass plate with microfibers was processed by plasma for 1 min to obtain a microfiber network with a hydrophilic surface. 10 wt% PVA solution (in deionized water) was cast on the glass plate with microfibers and followed to start the spinning-coating procedure. The spinning-coating rotation rates were 0.5, 1.0, 2.0, 3.0, and 5.0 k rpm, respectively. Subsequently, the above-mentioned PVA spinning-coated glass plate with microfibers was placed on a fried ice machine (CAUKD, CBJF–1DC450) for freeze–thaw processes of 4 cycles to obtain the microfiber composite hydrogel (PVA/MF-CH). PVA/MF/Gly-CH were prepared by soaking PVA/MF-CH in the glycerol or the mixture of glycerol and 0.9 wt% NaCl solution for 5 h. The mixtures of glycerol and 0.9 wt% NaCl solution were prepared by mixing the different volumes of glycerol and NaCl solution, designating as 2Gly 1NaCl, 1Gly 1NaCl, and 1Gly 2NaCl, respectively. Correspondingly, the microfiber composite hydrogels were named PVA/MF/Gly-CH with 2Gly 1NaCl, PVA/MF/Gly-CH with 1Gly 1NaCl, and PVA/MF/Gly-CH with 1Gly 2NaCl, respectively. PVA/MF-CH with 5% NaCl was prepared by soaking PVA/MF-CH in the 5 wt% NaCl solution for 5 h.

### Preparation of PVA/MF/Gly-CH-based Bioelectrode

PU microfiber networks were prepared by using the same parameters of the preparation procedure of the microfiber composite hydrogel as aforementioned, except that the electrospinning time was 5 min. Next, a layer of 50 nm Au was sputtered on the PU microfiber network to obtain the conductive microfiber network. Specific patterns were fabricated by using a mask during the sputtering process. The used spinning coating parameter was 1.0 k rpm, 10 s. A 4 freeze–thaw -cycle was used to obtain PVA/MF-CH-based bioelectrode. At last, PVA/MF/Gly-CH-based bioelectrode was obtained by soaking PVA/MF-CH-based bioelectrode in the glycerol or the mixture of glycerol and 5 wt% NaCl solution for 5 h.

### Characterizations

The morphology of the samples was performed on scanning electron microscopy (SEM, JSM-7001F). The optical images of microfiber networks were conducted on the Microscope (SOPTOP, CX40M). Uniaxial tensile measurements were carried out on an Instron 3365 (Instron, Norwood, MA, USA) universal materials tester at a constant moving speed of 20 mm min^−1^. The test gauges were cut into a rectangle of 2 cm × 0.5 cm. The electrochemical impedance was measured with a Gamry multichannel electrochemistry testing system (Interface 1000B and Reference 600 + , Gamry Instruments, Inc.). The potentiostatic EIS model was used to record the impedance of different frequencies and a voltage of 100 mV was applied. The surface roughness of the artificial skin covered with the microfiber composite hydrogel was measured with Surface Profiler (Veeco Dektak 150).

### EMG Measurements

In this work, we applied our PVA/MF/Gly-CH in two different ways. The first way is integrating it into the commercial EMG electrode by replacing its gel component, and two commercial EMG electrodes were applied as reference electrodes and GND electrodes, respectively. The second way is using our PVA/MF/Gly-CH-based bioelectrode as EMG electrodes to record biopotential signals. In this case, our n PVA/MF/Gly-CH-based bioelectrode was used as a testing electrode and a reference electrode, respectively, and the other two electrodes were employed by commercial electrodes. In another case, a tri-electrode system based on a patterned PVA/MF/Gly-CH-based bioelectrode (as shown in Fig. 5j later) was laminated on the skin to record EMG biosignals. For EMG measurements, three electrodes were attached to the forearm and the bicipital muscle, and EMG signals were recorded by multiplex bioelectric data acquisition equipment (ZJE-II) with a sampling rate of 500 Hz.

## Results and Discussion

### Design of Microfiber Composite Hydrogel

We introduce a simple method to fabricate microfiber composite hydrogel films with tailored thickness and mechanical properties, based on the combined fabrication procedure of the electrospinning technology and spin-coating method (Fig. 1b). Briefly, a PU microfiber network (fiber diameter 1.4 ± 0.5 μm) was electrospun onto a glass plate and then embedded into the PVA solution (10 wt%, in water) by spin-coating. Subsequently, the aforementioned microfiber-embedded PVA solution was placed on the frozen plate of an ice machine to freeze (− 20 °C, 30 min) and thaw (22 °C, 30 min) in the air multiple times (4, 6, and 8 cycles) to obtain the PVA/MF-CH. The obtained PVA/MF-CH presents a fibril-embedded porous structure, as shown in Fig. 1a. Figure 1c demonstrates the ECM-mimicked structure of the PVA/MF-CH, comprised of PU microfibers and PVA molecular chains. Through the freeze-thaw procedure, crystalline regions [43] and the inter-hydrogen bond [44] generate in some parts of the PVA molecular chains, induced by the growth of ice crystals, and thus form the physically crosslinked chain networks, illustrated in Fig. 1d. The PU microfiber network plays the role of the skeleton in the PVA/MF-CH, enhancing the system’s mechanical properties. Additionally, the hydroxyl group of PVA chains and the urethane group of PU microfibers have a strong hydrogen bond association that provides good interfacial linkage between microfibers and PVA [45], further improving the mechanical stability of the composite hydrogel system. The mechanically enhanced composite hydrogel enables the fabrication of a self-standing film with a thickness of < 7 μm (Fig. S1) and a high water content of > 90 %, which is difficult to obtain from a conventionally pure hydrogel system without sacrificing its water content. Contributed by the ultrathin configuration and ultrasoft characteristic, a PVA/MF-CH thin film (~ 7 μm) enables to realize seamless attachment onto the human skin (Fig. 1e) and the soft biological tissues with an irregular surface. Figure S2 displays the PVA/MF-CH attached to artificial leather mimicking skin, where the textures can be distinctly distinguished from the SEM image.

### Mechanical Performance of Microfiber Composite Hydrogel

**Fig. 2:**
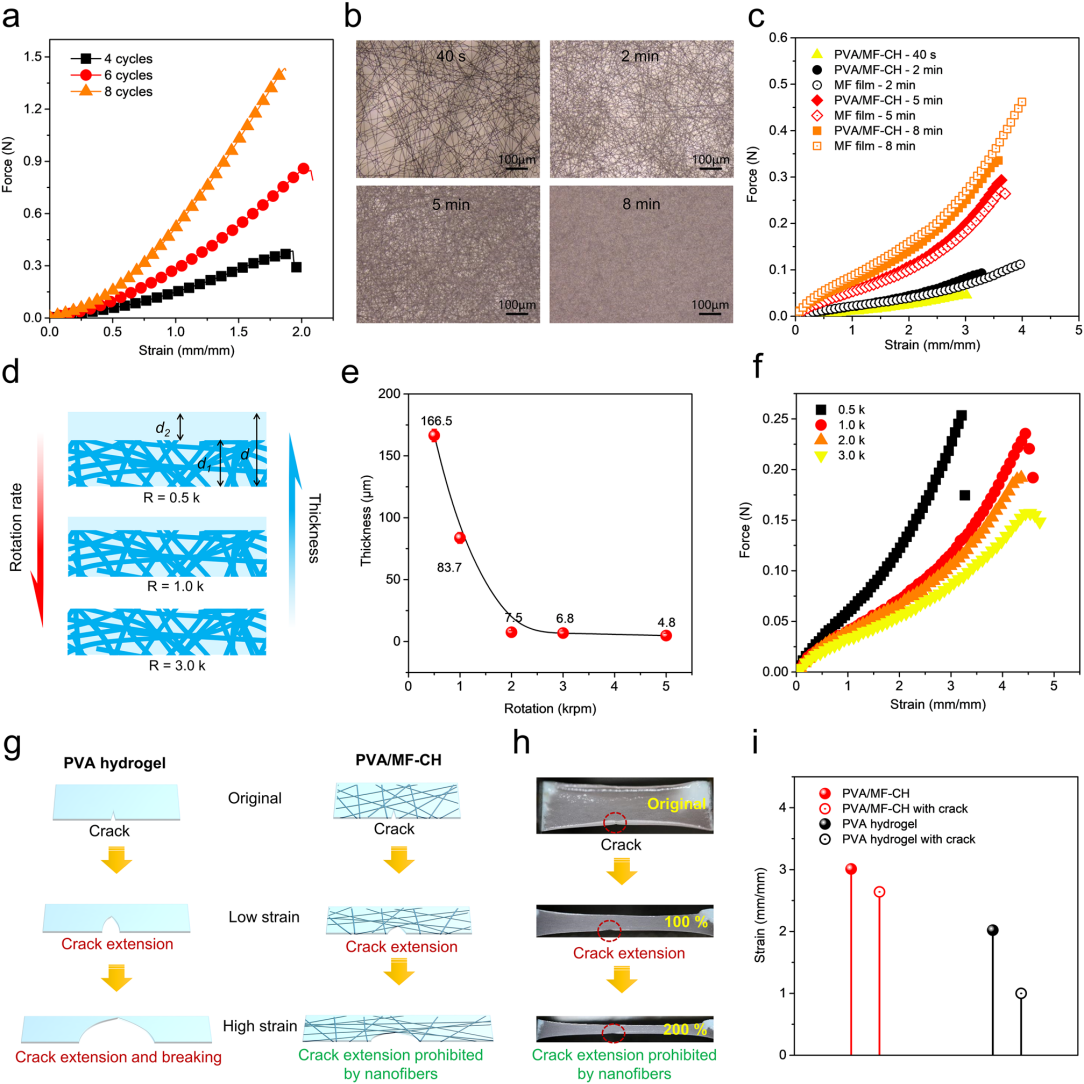
Mechanical properties of PVA/MF-CH. **a** Force-strain curve of the PVA hydrogel with different freeze-thaw cycles (4, 6, and 8). **b** Optical images of PU microfiber networks prepared with different electrospinning times (40 s, 2 min, 5 min, and 8 min). **c** Force-strain curves of PVA/MF-CH composed of PU microfiber networks with different electrospinning times (40 s, 2 min, 5 min, and 8 min) and PU microfiber networks with different electrospinning times of 2, 5, and 8 min. **d** Schematic thickness change of PVA/MF-CH with the increase of rotation rate. The thickness of the microfiber composite hydrogel (*d*) is comprised of the thickness of the microfiber network (*d*_*1*_) and the thickness of the PVA hydrogel over the microfiber network (*d*_*2*_) which can be controlled by the rotation rate during the spinning-coating procedure.** e** Thickness of PVA/MF-CHs processed under different rotation rates. **f** Force-strain curves of the PVA/MF-CH processed under different rotation rates of 0.5, 1.0, 2.0, and 3.0 k rpm. **g** Schematic anti-tearing behavior of microfiber composite hydrogel. **h** Digital images of PVA/MF-CH with a cut crack under different strains. **i** Maximum strains of PVA/MF-CHs and pure PVA hydrogels with and without a crack

The mechanical properties of the PVA/MF-CH can be tailored by the number of freeze-thaw cycles [45, 46]. The multiple freeze-thaw cycles promote more physical cross-links (including crystalline regions and hydrogen bonds) among PVA molecular chains, leading to enhanced strength (Fig. 2a) and Young’s Modulus (Fig. S3). In this work, the specimen (pure PVA hydrogel, 40 mm (length) × 10 mm (width) × 0.8 mm (thickness)) with 8 freeze-thaw cycles endured a high tensile force of ~ 1.5 N and possessed a Young’s Modulus of ~ 0.04 MPa. In a contrast, the specimen with 4 freeze-thaw cycles displayed a low tensile force of < 0.5 N and a Young’s Modulus of ~ 0.025 MPa. The PVA hydrogel samples prepared using less than 2 freeze-thaw cycles were too soft to form free-standing films. Although, more cross-links led to a drop in water content from 96.3 % ± 0.7 % (2 cycles) to 92.0 % ± 0.2 % (8 cycles), still the equilibrium water content was high.

The mechanical properties of the PVA/MF-CH are significantly influenced by the density of the microfiber network, which is tailored by controlling the electrospinning time. As shown in Fig. 2b, the density of microfiber networks increased as electrospinning time increased (electrospinning time 8 min), leading to high mechanical strength. The corresponding microfiber composite hydrogel showed a similar mechanical behavior (Fig. 2c), demonstrating the typically enhanced tensile force. A piece of ultrathin PVA/MF-CH (~ 7 μm) with high fiber density (electrospinning time of 8 min) can easily endure the 20 g weight as shown in Fig. S4. In contrast, the pure PVA hydrogel prepared under the same freeze-thaw condition was even not self-standing its own weight. Apart from the density of the microfiber network, the alignment of microfibers (Fig. S5) leads to enhanced mechanical properties with an anisotropic feature, illustrated in Fig. S6.

The thickness of PVA/MF-CH could be controlled by either changing the spin-coating speed or the concentration of the PVA solution and the thickness of the PVA/MF-CHs based on different preparation parameters are summarized in Table 1. As illustrated in Fig. 2d, using a low rotation rate (0.5 k rpm, 10 s) of spin-coating during preparation, the microfiber network was semi-embedded into the PVA hydrogel, resulting in a large thickness. Increasing the rotation rate of the spin-coating, the thickness of PVA/MF-CH dramatically declined from 166.5 μm (0.5 k rpm, 10 s) to 6.8 μm (3.0 k rpm, 10 s) and the microfiber network remains embedded in the PVA from the SEM image (Fig. S7). Further increasing the rotation rate to 5.0 k rpm (10 s), the thickness of PVA/MF-CH decreased to ~ 4.8 μm. The thicknesses of PVA/MF-CH fabricated in different rotation rates are illustrated in Fig. 2e and the corresponding images in Fig. S8. In addition, a thinner configuration (Fig. S9) merely of ~ 2 μm was obtained by using a 2.5 % PVA solution precursor. This indicated that the thickness of PVA/MF-CH was determined by the thickness of the microfiber network. As a result, the small thickness contributed to a weak tensile force (Fig. 2f). Moreover, our results demonstrated that the thickness of PVA/MF-CH was insignificantly affected by the amount of PVA solution used, as the superfluous PVA solution will remove during the spin-coating process. Whereas, too less amount of PVA solution used will result in the occurrence that PVA solutions are insufficient to cover all parts of the fiber networks (Fig. S10).

**Table 1:** Thickness of the PVA/MF-CHs based on different preparation parameters

Electrospinning parameters	Spin-coating parameters (k rpm, s)	Concentration of PVA solution (wt%)	Thickness (μm)
22 wt% PU solution Feeding rate 0.1 mm min^−1^ (5 mL syringe) Voltage 12 V Distance 15 cm Time 5 min	0.5, 10	10	166.5 ± 5.0
	1.0, 10	10	38.4 ± 4.1
	2.0, 10	10	7.6 ± 1.2
	3.0, 10	10	6.9 ± 1.6
	5.0, 10	10	4.8 ± 1.8
	3.0, 10	5	2.7 ± 1.6
	3.0, 10	2.5	1.7 ± 0.6

The nanofibrous structure also imparts PVA/MF-CH with very high puncture resistance. As shown in Fig. S11, the PVA/MF-CH endured large puncture stress from a pin without breaking. Another interesting merit of the PVA/MF-CH is its anti-tear behavior. As depicted in Fig. 2g, the high resistance of PVA/MF-CH to tearing is reasonable since the embedded PU microfibers create large force transfer lengths through high fiber/matrix modulus ratios and thus block cracks from further propagation. Figure 2h demonstrates the excellent anti-tearing performance of our PVA/MF-CH. The strain at break of PVA/MF-CH was up to 264 % (88 % of that without crack), and the pure PVA hydrogel was only extended to 100 % (50 % of that without crack) in contrast (Fig. 2i).

### Anti-dehydration Behavior and Conductivity

**Fig. 3:**
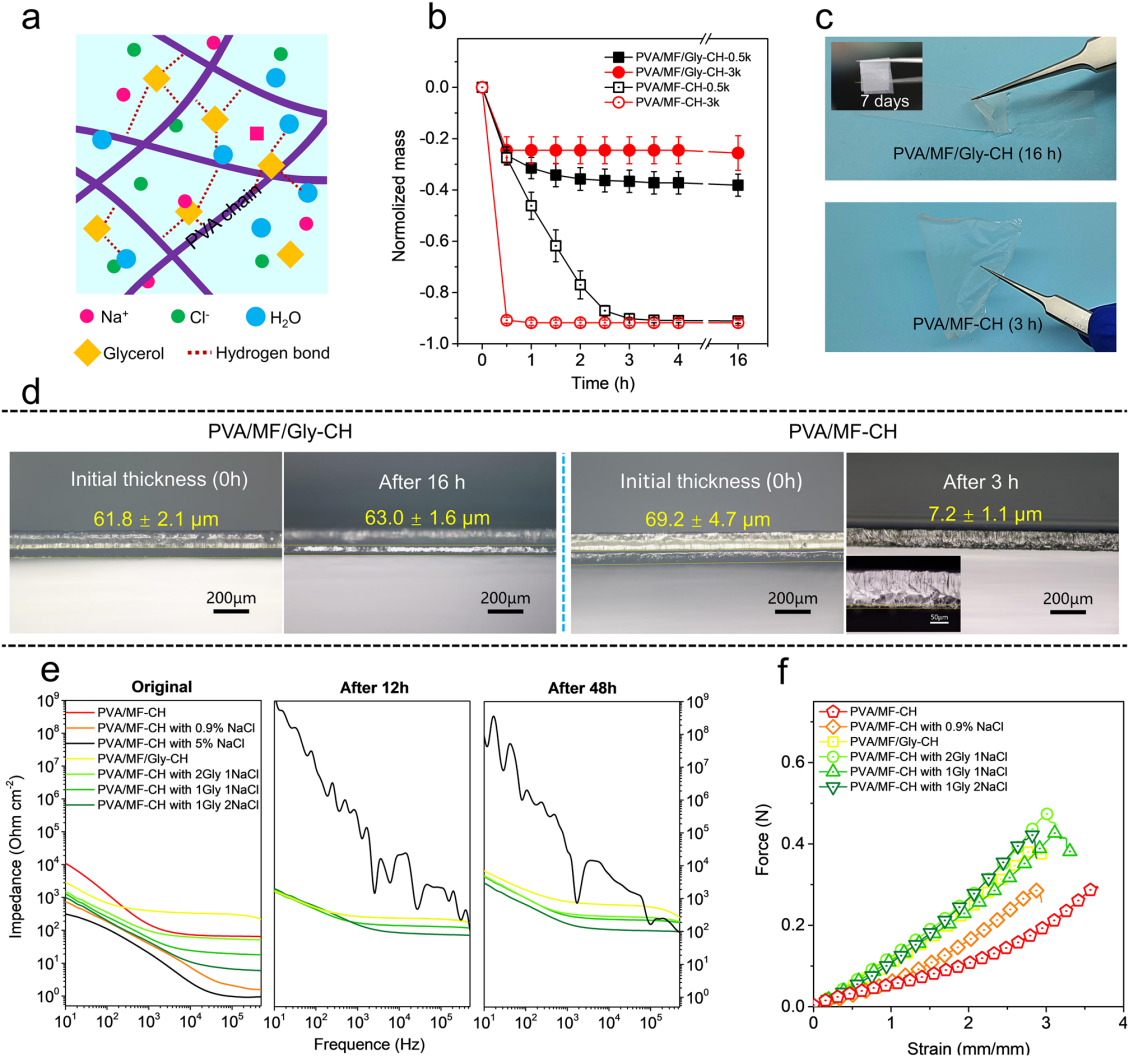
Dehydration behavior and conductivity. **a** Schematic structure and constituents of PVA/MF/Gly-CH with NaCl. **b** Mass change of PVA/MF/Gly-CH and PVA/MF -CH. **c** Digital images of PVA/MF/Gly-CH and PVA/MF-CH exposed in air for different time duration. The inserted picture is the PVA/MF/Gly-CH stored in the air for 7 days, which yet sustains its flexibility. **d** Optical images displaying the thickness change of PVA/MF/Gly-CH and PVA/MF-CH. **e** Electrochemical impedance of PVA/MF-CH with different amounts of glycerol and NaCl and corresponding electrochemical impedance after exposure in the air for 12 and 48 h. **f** Force-strain curve of PVA/MF-CH with different amounts of glycerol and NaCl

Compared to traditional bulk hydrogels, the ultrathin configuration imparts the PVA/MF-CH a large surface area exposed to its ambient surrounding, causing the issue of easy dehydration. The fast dehydration of hydrogel results in a dramatic increase in impedance in a short period. The incorporation of salts (such as LiCl, NaCl, and KCl) and other conductive fillers enables the decrease in the impedance of hydrogel, which is beneficial for the collection of high-quality biosignals when used as a bioelectrode. The anti-dehydration and impedance of the PVA/MF-CH were improved by soaking it in the mixture of NaCl/Glycerol. The glycerol in the PVA/MF-CH forms abundant hydrogen bonds with water molecules and efficiently inhibits volatilization and crystallization of free water molecules [47], endowing the PVA/MF-CH with excellent anti-dehydration and anti-freezing behavior, illustrated in Fig. 3a. The PVA/MF-CH with glycerol (PVA/MF/Gly-CH) maintained its mass (Fig. 3b) and configuration for over 16 h in the air (22 °C, ~ 40 % humidity). The flexibility of the PVA/MF/Gly-CH was well preserved for 7 days (Fig. 3c). However, the PVA/MF-CH lost its inherent soft nature and solidify into a rigid thin dry film within only 3 h due to the fast evaporation of water. Correspondingly, the thickness of PVA/MF-CH dramatically decreased from ~ 70 (0 h) to ~ 7 (after 3 h) μm, due to the loss of water (Fig. 3d). As a result of the abundant hydrogen bonds induced by the incorporation of glycerol, the PVA/MF/Gly-CH sustained a stable configuration and maintained its thickness of ~ 60 μm. A small drop of mass in the beginning is due to the evaporation of the free water incorporated in the PVA/MF-CH. The dehydration is also influenced by the thickness of the microfiber composite hydrogel. As shown in Fig. 3b, the thinner PVA/MF-CH (~ 7 μm, preparation parameters: 3.0 k rpm, 10 s) lost all its water within 0.5 h. The flexibility of another intriguing merit induced by glycerol is its anti-freezing property of the PVA/MF/Gly-CH. The PVA/MF/Gly-CH preserved excellent flexibility at a temperature of − 20 °C. In contrast, the PVA/MF-CH lost its flexibility because of the formation of ice crystals (Fig. S12). However, the introduction of glycerol to the PVA/MF-CH resulted in a slight increase of impedance from ~ 55 (PVA/MF-CH) to ~ 65 (PVA/MF/Gly-CH) ohm cm^−2^ (10^5^ Hz), as shown in Fig. 3e (impedances under different Hz in Table S1), because the use of the glycerol/H_2_O binary solvent could reduce the ionization of the water [48]. To decrease the impedance of the PVA/MF/Gly-CH, different amounts of 0.9 wt% NaCl (DI water solution) were incorporated to enhance its ion conductivity. The impedance of PVA/MF-CH significantly decreases with the increase of NaCl. We further also used concentrated NaCl solution (5 wt%). The impedance of the PVA/MF-CH significantly decreased (~ 1 Ω cm^−2^). Additionally, as the above-mentioned, the introduction of glycerol imparts the PVA/MF/Gly-CH anti-dehydration effect, which is favorable for sustaining the impedance stability of the PVA/MF/Gly-CH. Only slight increases occurred on the PVA/MF/Gly-CH. In contrast, the PVA/MF-CH with 5 wt% NaCl held a dramatic impedance change, especially in low frequency, which increases about 8 power of magnitude in the frequency of 10 Hz. These results indicate that the electrical properties of microfiber composite hydrogels are tunable by introducing salt ions according to the practical requirement. Except for the introduction of conductive ions, other conductive additives, such as carbon nanotubes and conducting polymers, can effectively improve the conductivity of the microfiber composite hydrogel.

The abundant hydrogen bonds [47] introduced from the incorporation of glycerol improved the mechanical strength and Young’s Modulus of the PVA/MF/Gly-CH, but the maximum strain slightly decreased (Fig. 3f). Additionally, the incorporation of glycerol decreased the thickness of microfiber composite hydrogel (Fig. S13). The addition of NaCl showed an insignificant influence on the improvement of mechanical properties.

### Tunable Conformability and Flexibility in a Broad Range

**Fig. 4:**
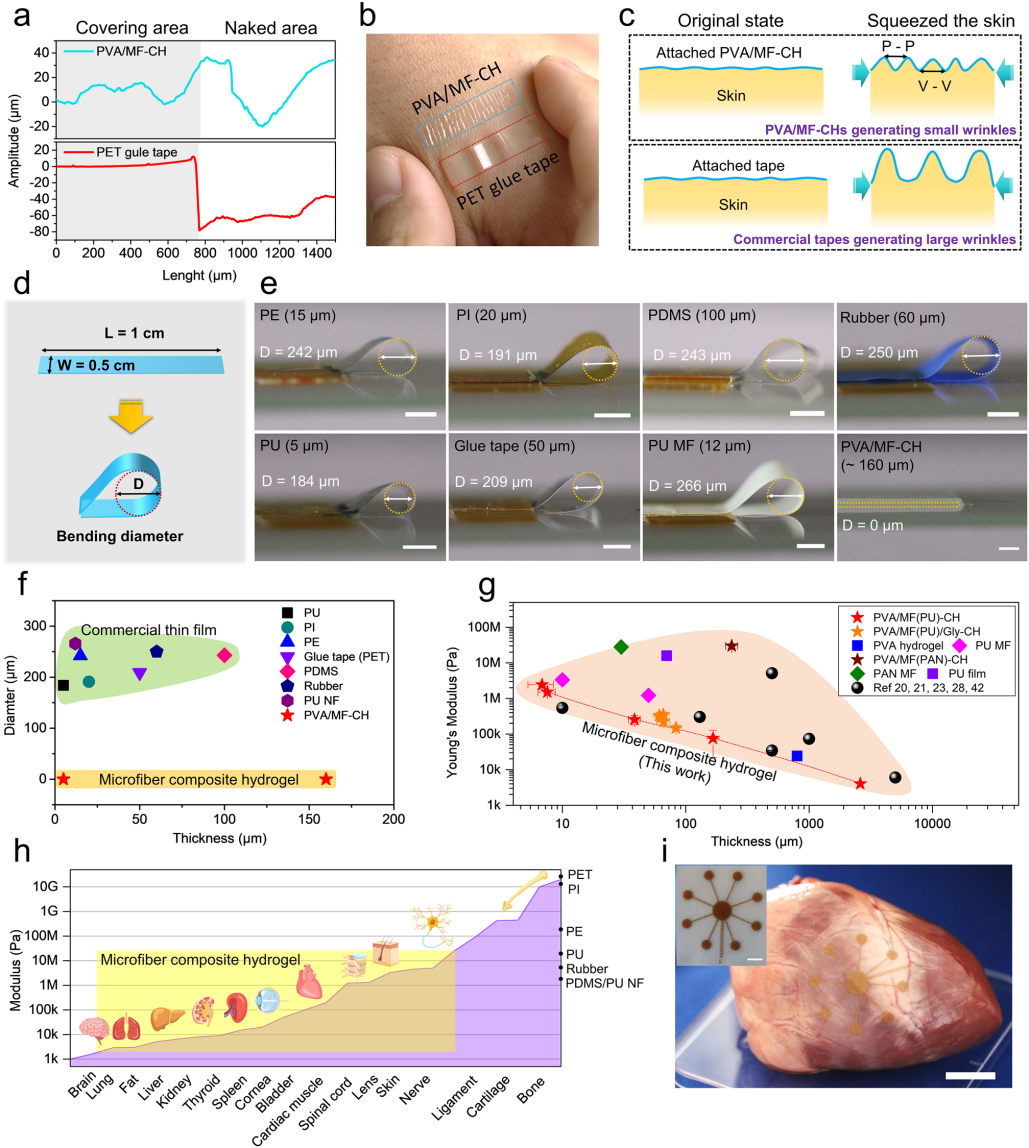
Tunable conformability and flexibility. **a** Surface roughness of the artificial skins. **b** Digital image displaying wrinkles generated from the PVA/MF-CH and PET glue tape induced by squeezing the skin. **c** Schematic wrinkle-generating mechanism of skin covered by PVA/MF-CH and PET glue tape when squeezing. **d** Schematic diagram of a method to evaluate the softness of a material with a bending diameter (D). **e** Digital images of bending diameters generated from different materials. Specimen size: 1 cm (L) × 0.5 cm (W). **f** The diameter of the bending circle generated in different materials. P-P and V-V mean the distance between two peaks and two valleys, respectively. **g** Young’s Modulus and thickness of different materials in this work and previously published works. PAN: polyacrylonitrile. **h** Modulus matching range of our PVA/MF-CH with biological tissues and organs. **i** Digital image of a porcine heart with attaching PVA/MF-CH -based bioelectrode. Scale bar 2 cm. The inserted picture is the PVA/MF-CH -based bioelectrode. Scale bar 1 cm

The conformability of a material to a rough surface is highly relative to the modulus and thickness of the film and the effect of each parameter is monotonic: the membrane tends to better conform to the rough surfaces as the membrane modulus decreases and the membrane thickness diminishes. For example, a 5-μm-thick Ecoflex membrane leads to intimate contact with the skin, but 36- and 100-μm-thick ones don’t [12, 26]. In addition, the stiffer the film, the smaller the critical thickness must be to realize full conformability. Compared to the conventional polymer films (modulus ~ GPa), such as Parylene, polyethylene terephthalate (PET), and polyimide (PI), the PVA/MF-CHs present excellent softness, which is favorable for realizing a seamless attachment to the rough surface. The skin conformability of the PVA/MF-CH (~ 80 μm) is evaluated by attaching it to the artificial skin and a thinner PET glue tape (50 μm) is used for comparison. The PVA/MF-CH demonstrated intimate contact with the artificial skin (Fig. S14) and similar results were also obtained from the samples attached to the human skin (Fig. S15). The microfiber composite hydrogel softly covers glyphic lines without forming any air gap. By contrast, air gaps were observed between the PET glue tape and the valleys of the artificial skin due to its large stiffness. The surface of the artificial skin covered with the PVA/MF-CH presented a similar rough surface profile as of naked areas (Fig. 4a). However, the surface amplitude completely disappeared in those areas covered by PET glue tape, demonstrating the rigid material's poor conformability with rough surfaces. Furthermore, the PVA/MF-CH possessed an excellent seamless attaching ability to various surface roughness, even to those objects with the millimeter scale amplitude, which is difficult to realize by using conventional plastic thin films (Fig. S16). Such an excellent conformal capability of the PVA/MF-CH is attributed to its ultrasoft nature, induced by the thin configuration and low modulus. Moreover, the PVA/MF-CH allows various deformations to occur on the human skin, such as squeezing, stretching, and bending deformations, indicating its excellent conformability and flexibility (Fig. S17). However, the rigid PET glue tape demonstrated limited deformable ability, caused by modulus mismatch between the skin and the attaching film. Squeezing the skin-attached PVA/MF-CH generated fine wrinkles (~ 1 mm, Fig. S18) and sustained the seamless attachments, as depicted in Fig. 4b, c. The rigid PET glue tape generated large wrinkles (~ 7 mm, Fig. S18) due to its large Young’s Modulus. We also evaluated the softness of the PVA/MF-CH with a bending diameter, which is described in Fig. 4d. Benefiting from its ultrasoft nature, the PVA/MF-CH displayed an unobservable bending angle (~ 0°) even though the specimen with a large thickness of ~ 160 μm (Fig. 4e). In contrast, conventional thin films which are often used as the substrates of flexible electronics presented a typical bending circle with diameters of ~ 200 μm induced by their large modulus. As illustrated in Fig. 4f, the PVA/MF-CHs in comparison with conventional polymer films hold an imperceptibly bending angle regardless of the thickness (~ 5–160 μm) due to its ultrasoft feature, indicating that our PVA/MF-CH is an ideal candidate for fabricating attachable bioelectronics. Even though the incorporation of glycerol slightly enhances the modulus as shown in Fig. 3f, which is adverse to achieving conformal contact, the conformability of PVA/MF/Gly-CHs to the skin (Fig. S19) is almost not affected by such a slight increase in modulus.

More significantly, our microfiber composite hydrogels hold tailored modulus by controlling their components of fiber, additives (with/without glycerol and salts), and thickness in a broad range, as shown in Fig. 4g, and even achieve smaller thicknesses and lower modulus than previous hydrogels [20, 21, 23, 28, 42], which impart better conformability. The feature of tunable modulus in a broad range (from kPa to tens of MPa) allows our microfiber composite hydrogel with a modulus match with most biological tissues and organs [9] (Fig. 4h), which is difficult to achieve from conventional thin films, such as PU, PI, polyethylene (PE), and polydimethylsiloxane (PDMS). Figure 4i displays a bioelectrode based on our PVA/MF-CH, which demonstrates excellent attaching and conformal capability to living biological tissues. However, its performance as implantable bioelectronics should be investigated in the future.

### Monitoring of EMG Biosignals

**Fig. 5:**
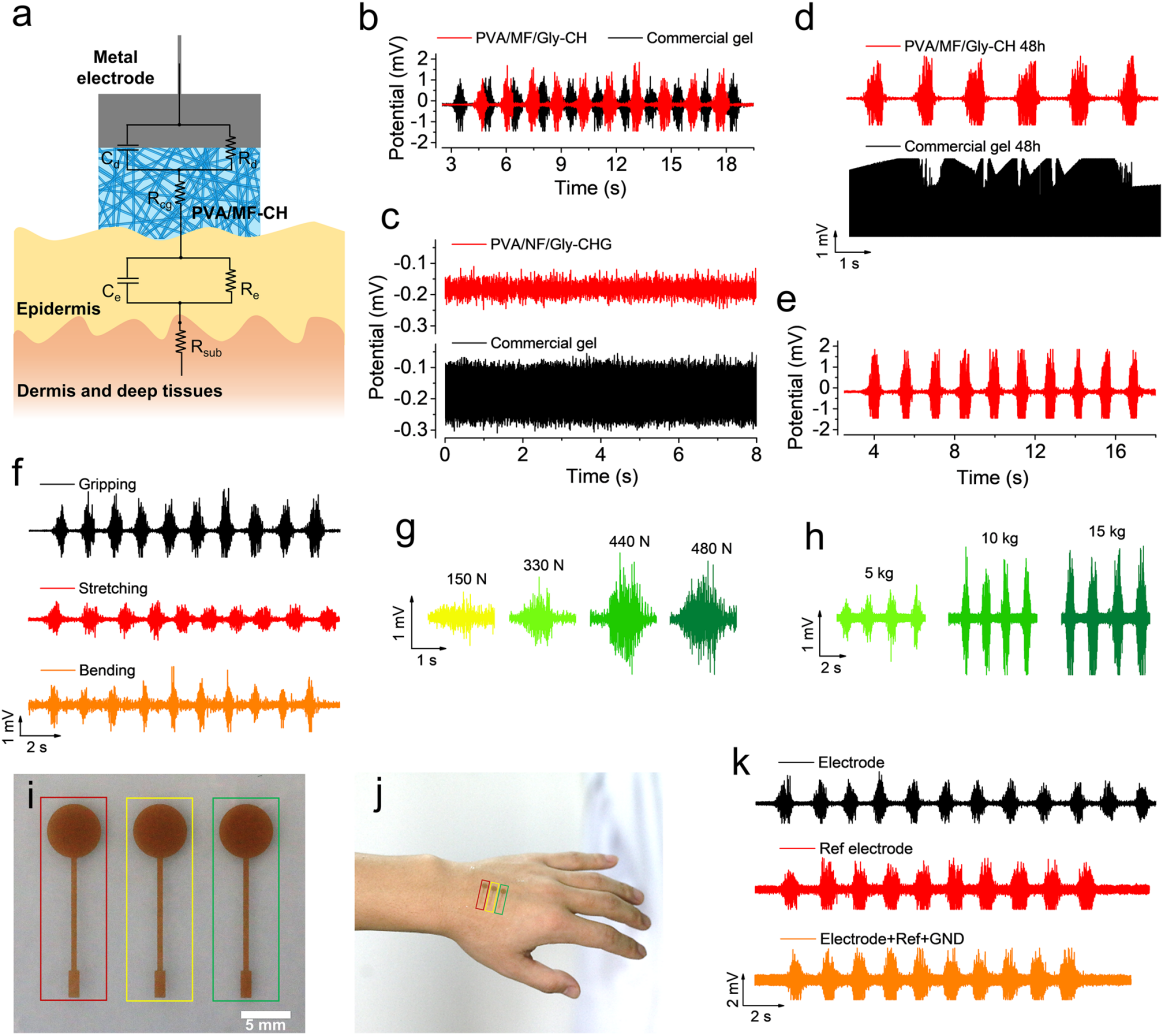
Monitoring of EMG biosignals. **a** Schematic of the equivalent circuit model used for monitoring EMG biosignals. At the electrode level (top three elements): *R*_d_ is the charge-transfer resistance, *C*_d_ is the double-layer capacitance, and *R*_cg_ is the resistance of our composite gel. At the skin level (bottom three elements), *R*_e_ and *C*_e_ are the epidermal resistance and capacitance, respectively, and *R*_sub_ is the resistance of the dermis and deep tissues. **b** Performance comparison of EMG biosignals collected by the electrode composed of our PVA/MF/Gly-CH and commercial gel. **c** The background noises of the electrode composed of our PVA/MF/Gly-CH and commercial gel. **d** Performance comparison of the electrode composed of our PVA/MF/Gly-CH and commercial gel for the monitoring EMG biosignals after 48 h. **e** Performance of the electrode composed of our PVA/MF/Gly-CH for the monitoring EMG biosignals after 7 d. **f** EMG biosignals of the forearm are generated from different gestures. **g** EMG biosignals of the forearm are generated from different gripping forces. **h** EMG biosignals of the bicipital muscle of the arm lifting the different masses of the object. **i** A tri-electrode system comprised of the PVA/MF/Gly-CH for the monitoring of EMG biosignals. (Electrode in red rectangle, GND in yellow rectangle, and Ref electrode in green rectangle) **j** Digital image of the hand with attached a tri-electrode system comprised of the PVA/MF/Gly-CH. **k** EMG biosignals of the forearm collected by our PVA/MF/Gly-CH-based bioelectrode

The simplified equivalent circuit model [49] describes a metal-gel electrode interfacing with the epidermis and subcutaneous skin layers, as depicted in Fig. 5a. The total impedance of this equivalent circuit model is expressed by Eq. (1):

1 $${Z}_{\mathrm{t}}={R}_{\mathrm{sub}}+ \left[\frac{{R}_{\mathrm{e}}}{1+ j\omega {C}_{\mathrm{e}}{R}_{\mathrm{e}}}\right]+ {R}_{\mathrm{cg}}+ \left[\frac{{R}_{\mathrm{d}}}{1+ j\omega {C}_{\mathrm{d}}{R}_{\mathrm{d}}}\right]$$

where *j* is the imaginary unit, *ω* = 2π*f* is the angular frequency in rad s^−1^, and *f* is the frequency in Hz. This model was chosen over the standard Randles cell to allow for more precise evaluation and comparison of the effects from the electrode versus skin elements. Specifically, the electrode elements include: (i) *R*_cg_, the composite gel resistance, (ii) *R*_d_, the charge-transfer resistance and (iii) *C*_d_, the capacitance of the double-layer between the metal electrode and the composite gel. The representative skin elements include: (i) *R*_sub_, the subcutaneous resistance of dermis and deep tissues, (ii) *R*_e_, the epidermal layer resistance, and (iii) *C*_e_, the epidermal capacitance.

Biopotentials are typically weak, ranging from several micro-volts to several millivolts. Specific to the cut-and-pasted large-area epidermal electrodes without interconnect encapsulation, the signals are further compromised by the dimensionless geometric parameter [50]. As a result, the background noise could become problematic. The thin configuration of gelled electrolytes contributes to lower *R*_cg_, and leads to a lower impedance of the whole system (Fig. S21), improving the SNR of the electrode. As shown in Fig. 5b, the electrode comprised of PVA/MF/Gly-CH displayed a similar biopotential amplitude of muscle activations with that recorded with a commercial gel electrode in the same region. Significantly, our PVA/MF/Gly-CH electrode recorded lower background noise (Fig. 5c), demonstrating an excellent SNR. Specific to the electrode comprised of the PVA/MF/Gly-CH, another distinct merit is the capability of use for the long term. As shown in Fig. 5d, the electrode comprised of the PVA/MF/Gly-CH sustain monitoring EMGs in high quality for 48 h in ambient air. In contrast, the commercial gel electrode lost its functionality caused by the dehydration of the gel. Moreover, the recording of high-quality EMGs were even possible after storage in the air over 10 days (Fig. 5e) with our PVA/MF/Gly-CH electrode, demonstrating the capability for long-term application as the new wearable electronics.

Electrophysiological activation of a muscle initiates mechanical force production. Typically, the position and the moving performances of the human body are based on the activation of a single or a multitude of muscles, and thus, generate distinguishable EMGs. Thereof, EMGs can be used to monitor physical activities. Figure 5f displayed distinguishable EMGs generated from the gripping, stretching finger, and bending wrist gestures, respectively. In addition, EMGs reflect the degree of activation: the higher the EMG level, the more force is developed by the muscle [51, 52]. Figure 5g displayed the EMGs of the forearm of a male adult with different grasping forces, recorded by our PVA/MF/Gly-CH electrode. Figure 5h was characteristic EMGs of the bicipital muscle of the arm lifting the different masses of the object. These results demonstrated the feasibility of acquiring high-quality biosignals in different body parts by using our PVA/MF/Gly-CH electrode, which can be used to analyze the intensity of muscle activities in different body parts.

Our PVA/MF/Gly-CH enabled it to be applied for monitoring biopotential signals, separately, to replace the commercial gel electrode. A tri-electrode system comprised of the PVA/MF/Gly-CH was shown in Fig. 5i. Such a tri-electrode system presented ultrathin configuration and excellent flexibility, leading to intimate contact with human skin (Fig. 5j) when laminated, and sustained multiple detaching processes (Fig. S20).

Figure 5k displayed the EMGs recorded by using our electrode as a testing electrode, Ref electrode, and a tri-electrode system (electrode + Ref + GND), respectively. In three cases, clear and distinguishable EMGs were recorded of the gripping gesture. However, it is worth noting that the EMGs recorded with the tri-electrode system presented relatively higher background noises, probably resulting from the poor adhesion between the electrode and human skin.

Since external pressure can induce resistance change of a hydrogel caused by the diminution of thickness, the PVA/MF/Gly-CH is promising for constructing ultrathin electronic-skin pressure sensors. We show a prototype of a pressure sensor (Fig. S22) by simply integrating double layers of the PVA/MF/Gly-CH-based electrode, which could be applied to detecting the force variations. Typically, the hydrogel-type sensor presents a long relaxation time to recover its performance. The incorporation of salt ions can effectively shorten the hydrogel sensor’s relaxation time. In our case, we observed that the pure PVA/MF/Gly-CH without NaCl held a relaxation time of over 1 min. The relaxation time of the PVA/MF/Gly-CH processed with 5 wt% NaCl significantly decreased to as low as ~ 5 s. The feature of a tailored relaxation time endows our microfiber composite hydrogel with a high possibility to construct a matrix sensor for monitoring object movement trace. Moreover, the prototype sensor holds a good capability to monitor the pressure in various frequencies and displayed a good working duration. In contrast to its pressure sensitivity, our PVA/MF/Gly-CH presented another merit of strain insensitive feature and even sustained multiple stretches for a long time.

## Conclusions

In this work, we develop a novel strategy to fabricate ultrathin microfiber composite hydrogel by combining electrospinning with spinning-coating. The microfiber composite hydrogel exhibited high tensile strength, modulus, and flexibility. The embedded microfiber network endowed microfiber composite hydrogel with prominent anti-tearing performance. The ultrathin configuration and ultrasoft nature allow it seamlessly attached to various rough surfaces. Moreover, The microfiber composite hydrogel presented mechanical features that matched modulus with almost all biological tissues. By the incorporation of glycerol, the microfiber composite hydrogel leads to anti-dehydration in a long term. Based on the aforementioned features, the EMG electrode composed of our microfiber composite hydrogel demonstrated excellent performance in EMG biosignal monitoring. Our strategy to fabricate ultrathin hydrogel will promote the progress of hydrogel bioelectronics, especially for the development of flexible electronics based on hydrogel films.

### Supplementary Information

Below is the link to the electronic supplementary material.

Supplementary file1 (PDF 1613 KB)
