# Supplementary material for: Biological Tissue-Inspired Ultrasoft, Ultrathin, and Mechanically Enhanced Microfiber Composite Hydrogel for Flexible Bioelectronics
Source: Nanomicro Lett. 2023 May 28;15:139. doi: 10.1007/s40820-023-01096-4 (PMC10225432; doi:10.1007/s40820-023-01096-4)
Supplement: Supplementary file 1 — Supplementary file1 (PDF 1613 KB) [file 40820_2023_1096_MOESM1_ESM.pdf]

Supporting Information for

Biological Tissue-Inspired Ultrasoft, Ultrathin, and Mechanically Enhanced Microfiber Composite Hydrogel for Flexible Bioelectronics

Qiang Gao[1], Fuqin Sun[1], Yue Li[1], Lianhui Li[1], Mengyuan Liu[1], Shuqi Wang[1], Yongfeng Wang[1], Tie Li[1], Lin Liu[1], Simin Feng[1], Xiaowei Wang[1], Seema Agarwal[2], Ting Zhang[1,3,4,]*

[1] i-Lab, Nano-X Vacuum Interconnected Workstation, Key Laboratory of Multifunction Nanomaterials and Smart Systems, Suzhou Institute of Nano-Tech and Nano-Bionics (SINANO), Chinese Academy of Sciences (CAS), Suzhou, Jiangsu, 215123, P. R. China

[2] Bavarian Center for Battery Technology (BayBatt), Bayreuth Center for Colloids and Interfaces, and Bavarian Polymer Institute, Macromolecular Chemistry II, Department of Chemistry, University of Bayreuth, Universitätsstrasse 30, Bayreuth, 95440, Germany

[3] School of Nano-Tech and Nano-Bionics, University of Science and Technology of China, Hefei, Anhui, 230026, P. R. China

[4] Center for Excellence in Brain Science and Intelligence Technology, Chinese Academy of Science, Shanghai, 200031, P. R. China

* Corresponding author. E-mail: tzhang2009@sinano.ac.cn (Ting Zhang)

Supplementary Figures and Tables

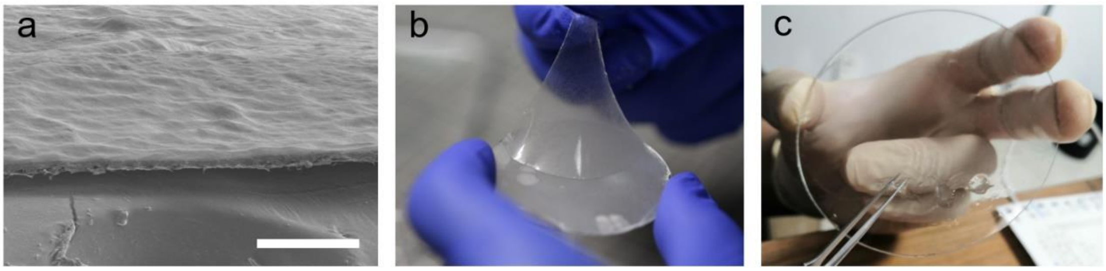

Fig. S1 a SEM image displayed the cross-section of PVA/MF-CH. **b** Digital image of stretched PVA/MF-CH. The microfiber network was used with one of electrospinning 5 min. The spinning coating parameters were used as follows, rotation rate 1.0 k, time 10 s. The freeze-thaw cycle was 4 times. **c** Digital image of The PVA hydrogel processed with the same parameters with PVA/MF-CH in **b**.

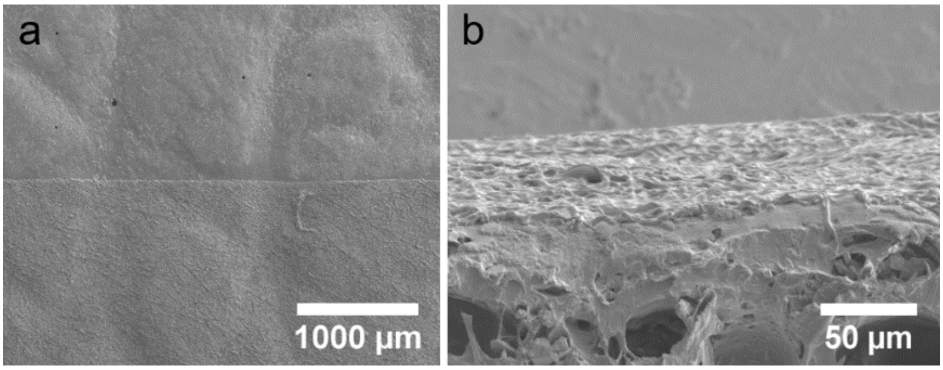

Fig. S2 SEM images of artificial skin covered by PVA/MF-CH. **a** surface morphology, **b** cross-section

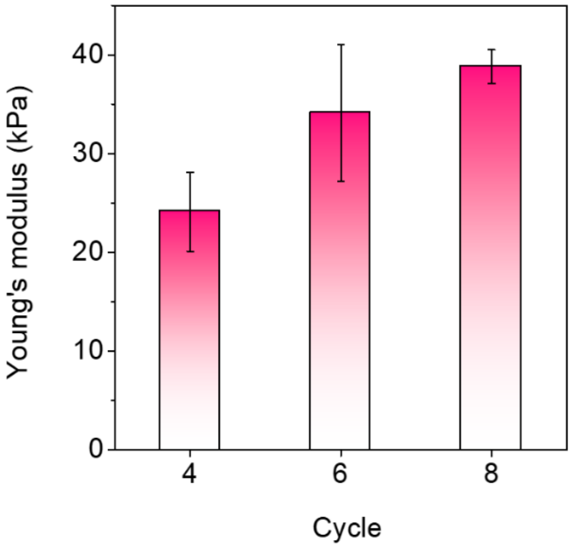

Fig. S3 Young's modulus of PVA hydrogel with different freeze-thaw cycles

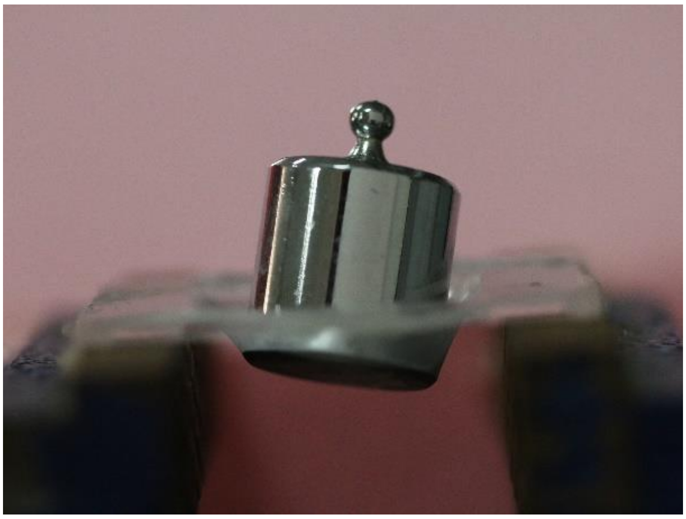

Fig. S4 Digital image of PVA/MF-CH (thickness of ~ 7 μm) holding an object of 20 g. The microfiber network was used with one of electrospinning for 8 min and the freeze-thaw cycle was 4 times

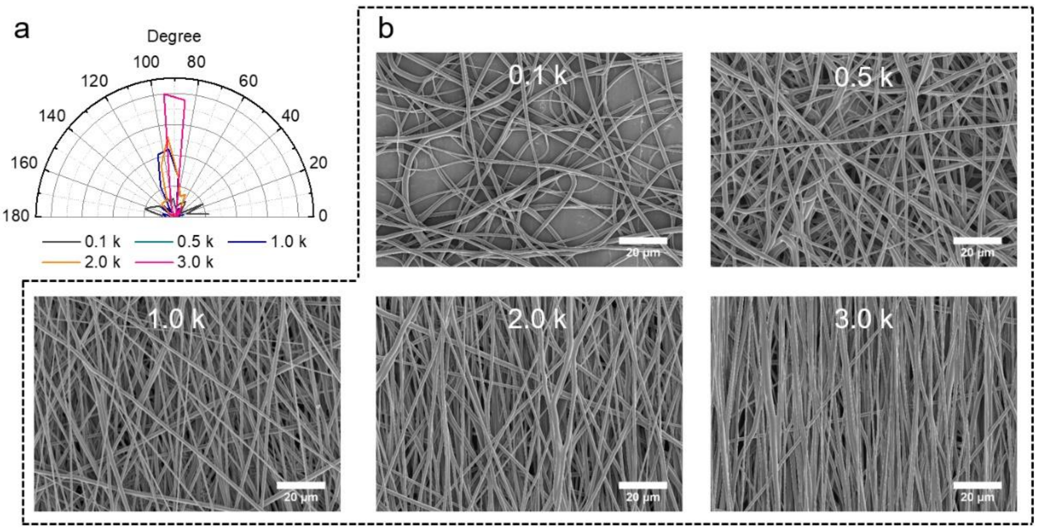

Fig. S5 a Anisotropic properties of microfiber networks collected with different rotation rates. a Statistic of microfiber orientation. 100 fibers were randomly selected from SEM images with Image J software. **b** SEM images of microfiber network collected with different rotation rates. Note that these microfibers were collected by a rotating drum instead of a plate collector. The orientation degree of the fiber alignment increases with the increase in rotation rate. The fibers collected under a low rotation rate (< 1.0 k) present random orientation. When the rotation rate of the drum increases to 3.0 k, the fibers are highly aligned in one direction.

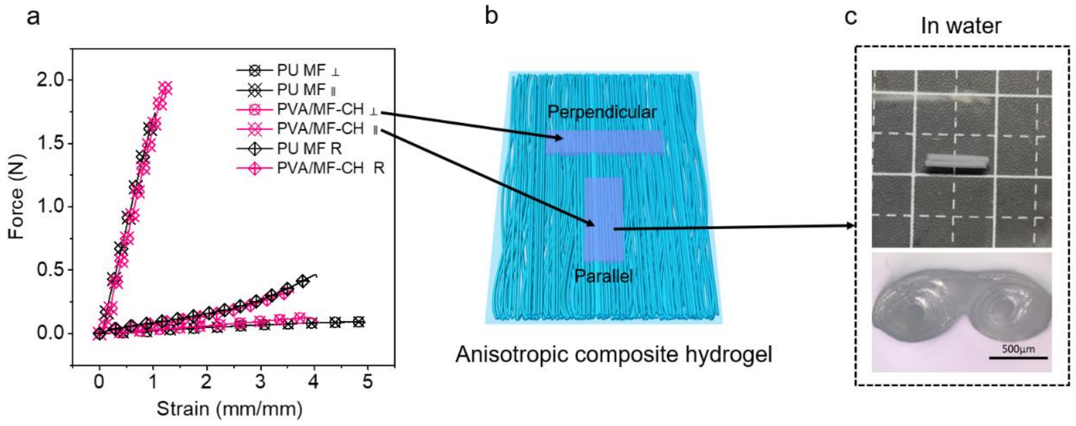

Fig. S6 a Force-strain curves of PVA/MF-CH and PU microfiber film with different fiber alignments. ⊥ means that the stretching direction is perpendicular to fiber orientation direction. ∥ means that stretching direction is parallel with fiber orientation direction. R means ramdom orientation. **b** Schematic diagram of the anisotropic composite hydrogel composed of aligned microfibers. **c** Self-curving behavior of the anisotropic composite hydrogel composed of parallel fiber orientation. The digital image (up) displayed a tube structure derived from a piece of dried anisotropic composite hydrogel (with a structure as schematic in b) reabsorbed water. Cross-sectional optical image (down) of the tube structure above-mentioned. The semi-embedded structured PVA/MF-CH with orientated fiber alignment leads to a self-crimping feature, caused by planar nonuniform swelling field [S1-S3] and interlayer

forces of PVA constituent in the microfiber semi-embedded structure when the dried PVA/MF-CH film is placed into a water bath.

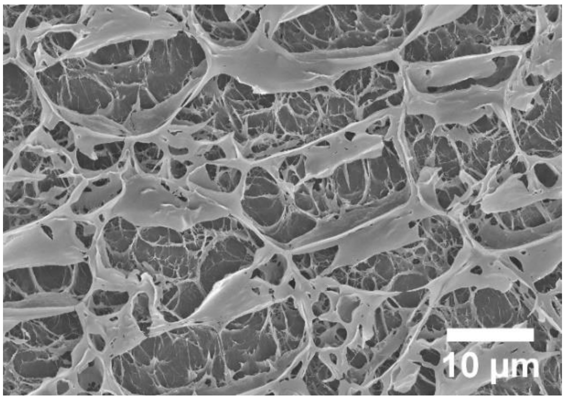

Fig. S7 SEM image of the surface morphology of PVA/MF-CH with 3.0 k (10 s) rotation process.

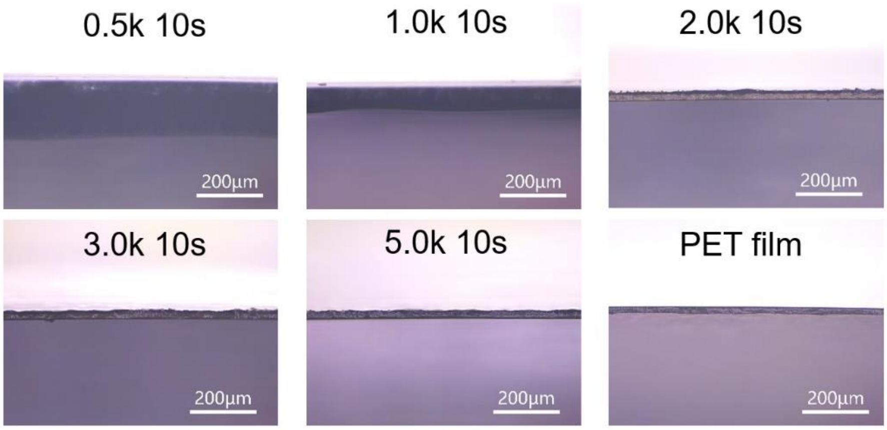

Fig. S8 Cross-sectional optical images of PVA/MF-CHs processed with different rotation rates attached to a PET film (50 μm)

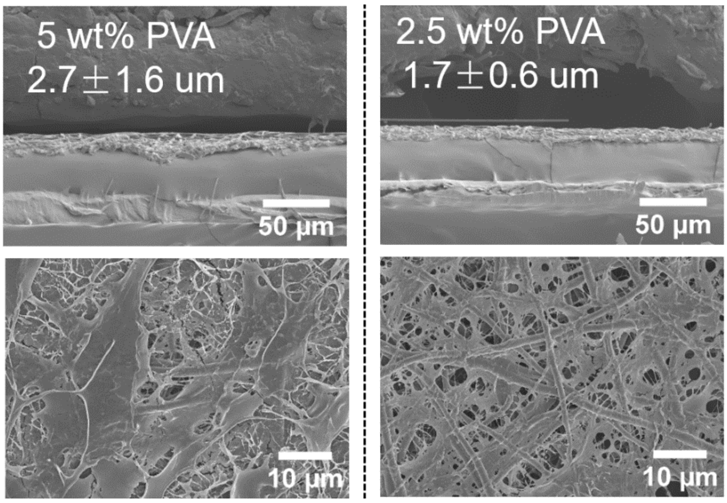

Fig. S9 The SEM images of PVA/MF-CHs derived from different concentrations of PVA solution. Up ones: cross-section; down ones: surface morphology

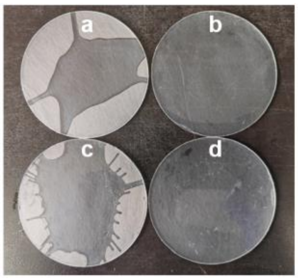

Sample	Amount of PVA / mL	Rotation rate / krpm	Rotation time / s	PVA covering area	Thickness / μm
a	1	0.5	10	44%	-
b	3	0.5	10	100%	~ 160 μm
-	5	0.5	10	100%	~ 160 μm
c	1	3.0	10	65%	-
d	3	3.0	10	100%	~ 7 μm
-	5	3.0	10	100%	~ 7 μm

Fig. S10 Effect of the amount of PVA solution used on microfiber composite hydrogels. Optical images of microfiber composite hydrogels with different amounts of PVA solution after spin-coating process (left) and their corresponding preparation parameters (right table). The diameter of the glass plate is 3 inches

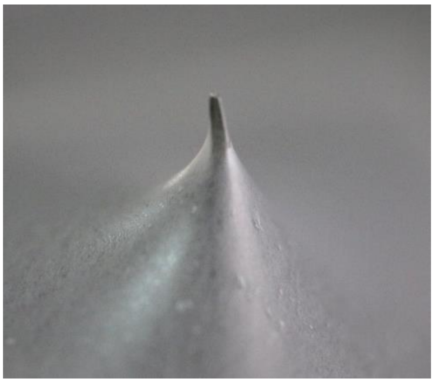

Fig. S11 Digital image displays the puncturing resistance of PVA/MF-CH

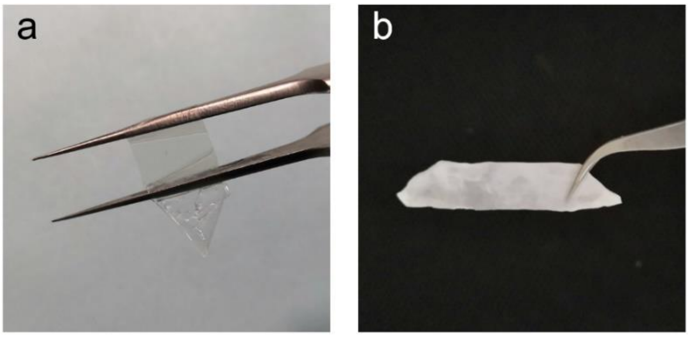

Fig. S12 Freezing behavior of PVA/MF/Gly-CH (**a**) and PVA/MF-CH (**b**)

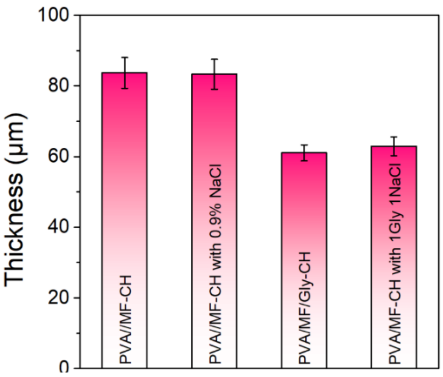

Fig. S13 Thickness of PVA/MF-CH contented of different constituents. The used concentration of NaCl was 0.9 wt%

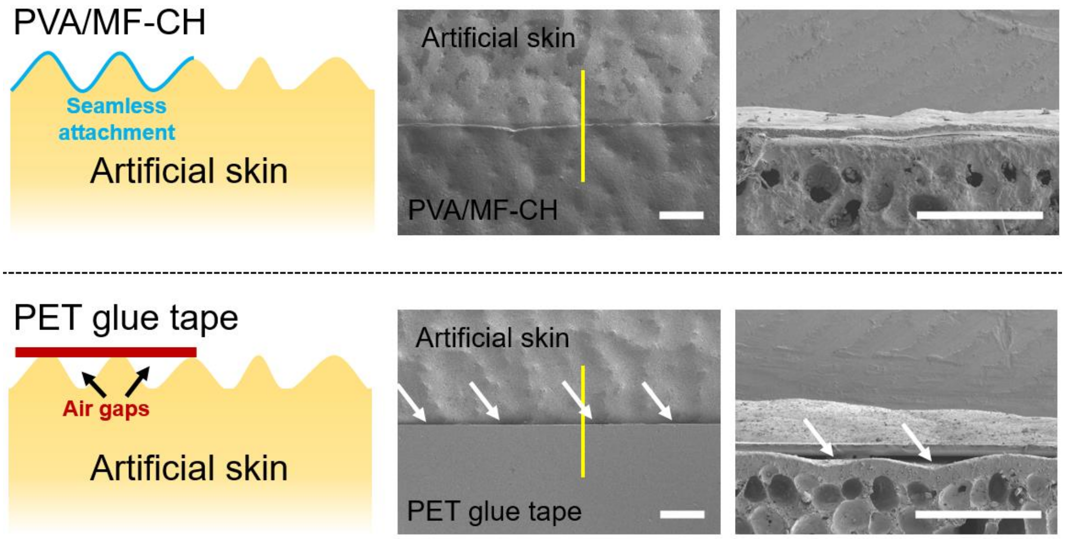

Fig. S14 Schematic diagrams and SEM images of the artificial skins covered by PVA/MF-CH and PET glue tape, respectively. Arrows point to air gaps. Yellow lines are the measuring regions of Fig. 4a in the main text. All scale bars are 500 μm.

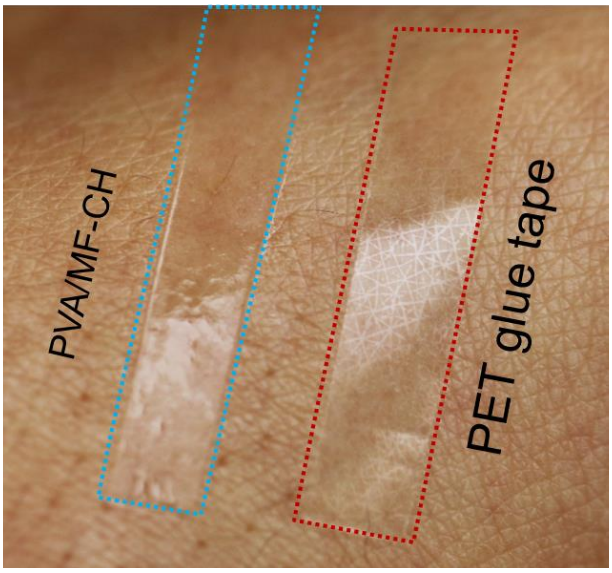

Fig. S15 Digital image of skin covered by PVA/MF-CH and PET glue tape

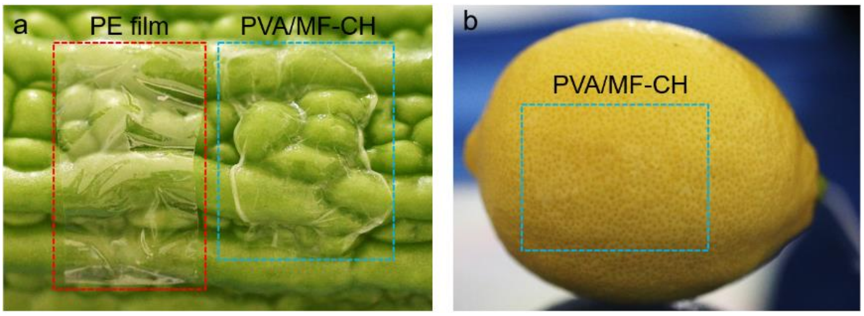

Fig. S16 Conformal behavior of PVA/MF-CH (~ 80 μm) with objects possessing different surface roughness. Attaching to the surface of a Momordica charantia (**a**) and a lemon (**b**). A PE film (20 μm) is as a contrast

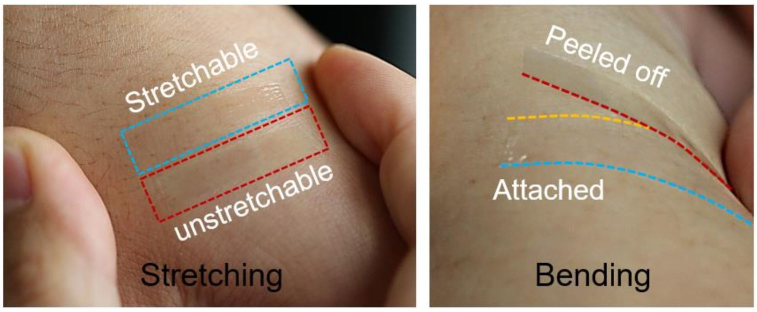

Fig. S17 Digital images of PVA/MF-CH (in the blue area) under deformation behaviors induced by stretching and bending the local skin. A PET glue tape (in the red area) was used as a contrast.

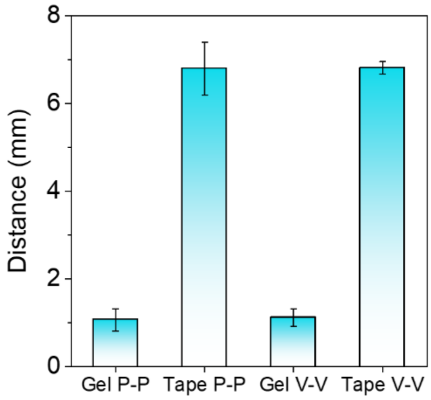

Fig. S18 Distance between wrinkles of PVA/MF-CH and PET glue tape. Here Gel presents the PVA/MF-CH and the Tape is the PET glue tape of 50 μm.

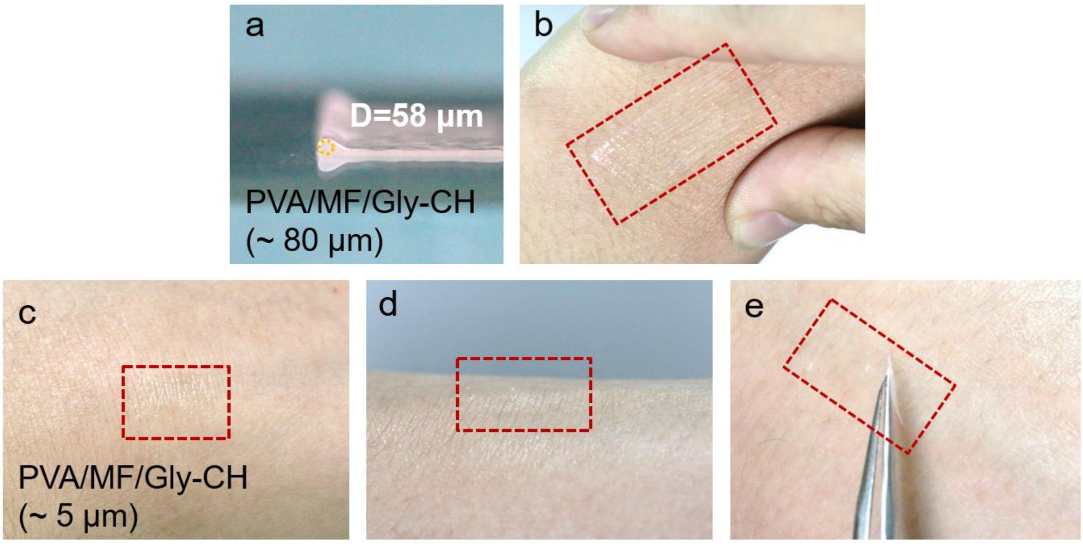

Fig. S19 a Digital images of bending diameters generated from a PVA/MF/Gly-CH with a thickness of ~ 80 μm. Specimen size: 1 cm (L) × 0.5 cm (W). **b** Digital image displaying wrinkles generated from a PVA/MF/Gly-CH with a thickness of ~ 80 μm induced by squeezing the skin. **c** (top view) and **d** (side view) Digital images of a PVA/MF/Gly-CH with a thickness of ~ 5 μm attached to the skin. **e** Digital image displaying a PVA/MF/Gly-CH with a thickness of ~ 5 μm detached from the skin

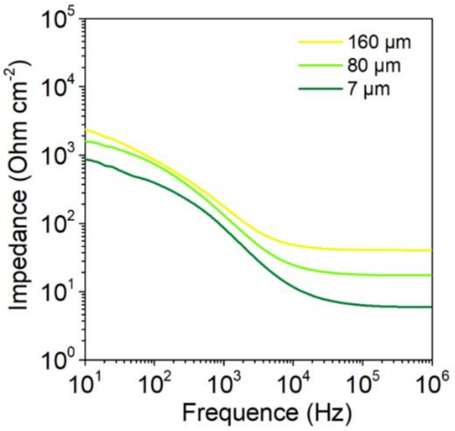

Fig. S20 Electrochemical impedance of PVA/MF-CH of different thicknesses

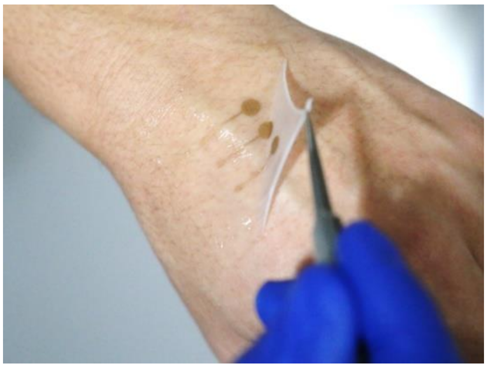

Fig. S21 Digital image of detaching PVA/MF-CH-based bioelectrode from the skin

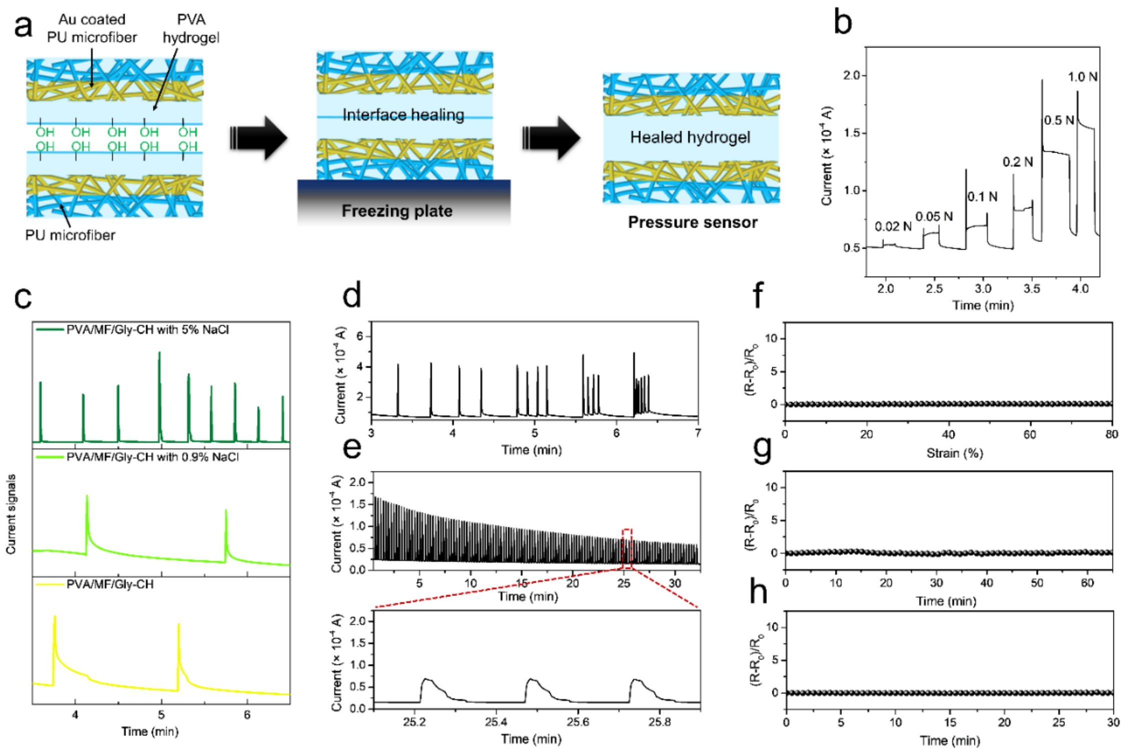

Fig. S22 a Schematic fabrication of a pressure sensor based on PVA/MF/Gly-CH-based bioelectrode. **b** The response of the pressure sensor based on PVA/MF/Gly-CH-based

bioelectrode to different loads. **c** The relaxation time of PVA/MF/Gly-CH-based bioelectrode with different concentrations of NaCl. **d** Sensing behavior of the pressure sensor based on PVA/MF/Gly-CH-based bioelectrode pressed by a finger at different rates. **e** Performance durability of the pressure sensor based on PVA/MF/Gly-CH-based bioelectrode. **f** Resistance change of the pressure sensor based on PVA/MF/Gly-CH-based bioelectrode under different strains. **g** Resistance stability of the pressure sensor based on PVA/MF/Gly-CH-based bioelectrode: 10% strain, 100 cycles. **h** Resistance stability of the pressure sensor based on PVA/MF/Gly-CH-based bioelectrode: holding 10% strain for 30 min

Table S1 Impedance of microfiber composite hydrogels in different time duration

Sample	Time	Frequency / Hz				
		10^1	10^2	10^3	10^4	10^5
PVA/MF-CH	original	10950	1292	151.7	73.6	66.5
PVA/MF-CH with 0.9 % NaCl		743.1	184.7	39.1	7.4	2.1
PVA/MF-CH with 5 % NaCl		314.2	110.4	20.5	2.6	1.0
PVA/MF-CH with Gly		2889	678.5	396.2	334.8	306.3
PVA/MF-CH with 2Gly 1 NaCl		1501	352	96.9	62.4	54.3
PVA/MF-CH with 1Gly 1 NaCl		1300	255.8	59.5	24.3	19.4
PVA/MF-CH with 1Gly 2 NaCl		1014	198.1	44.6	10.9	6.7
PVA/MF-CH with 5 % NaCl	12 h	2.0×10^9	7.0×10^6	371800	21100	2057
PVA/MF-CH with Gly		1628	502.4	276.7	248	229.1
PVA/MF-CH with 2Gly 1 NaCl		1754	522.4	277.8	247.8	230.8
PVA/MF-CH with 1Gly 1 NaCl		1588	453.8	177.8	147.1	136.8
PVA/MF-CH with 1Gly 2 NaCl		1967	556.2	156.2	88.8	78.8
PVA/MF-CH with 5 % NaCl	48 h	1.0×10^8	2.9×10^6	19960	5494	131.3
PVA/MF-CH with Gly		6959	1833	813.6	654.1	555.8
PVA/MF-CH with 2Gly 1 NaCl		4944	1030	350.2	288.3	265.6
PVA/MF-CH with 1Gly 1 NaCl		4687	1040	303.8	234.7	218.8
PVA/MF-CH with 1Gly 2 NaCl		3019	657	172.1	114.7	105.5

Supplementary References

[S1] L. Liu, A. Ghaemi, S. Gekle, S. Agarwal, One-component dual actuation: Poly (NIPAM) can actuate to stable 3D forms with reversible size change. Adv. Mater. **28**(44), 9792-9796 (2016). https://doi.org/10.1002/adma.201603677

[S2] L. Liu, S. Jiang, Y. Sun, S. Agarwal, Giving direction to motion and surface with ultra-fast speed using oriented hydrogel fibers. Adv. Funct. Mater. **26**(7), 1021-1027 (2016). https://doi.org/10.1002/adfm.201503612

[S3] T. Chen, H. Bakhshi, L. Liu, J. Ji, S. Agarwal, Combining 3d printing with electrospinning for rapid response and enhanced designability of hydrogel actuators. Adv. Funct. Mater. **28**(19), 1800514 (2018). https://doi.org/10.1002/adfm.201800514
